# Clinical and Mutation Analysis of Patients with Best Vitelliform Macular Dystrophy or Autosomal Recessive Bestrophinopathy in Chinese Population

**DOI:** 10.1155/2018/4582816

**Published:** 2018-10-18

**Authors:** Tingting Gao, Chengqiang Tian, Qinrui Hu, Zhiming Liu, Jimei Zou, Lvzhen Huang, Mingwei Zhao

**Affiliations:** ^1^Department of Ophthalmology, Peking University People's Hospital, Eye Diseases and Optometry Institute, Beijing Key Laboratory of Diagnosis and Therapy of Retinal and Choroid Diseases, College of Optometry, Peking University Health Science Center, Beijing, China; ^2^Department of Ophthalmology, The First Affiliated Hospital of Dalian Medical University, China; ^3^Chinese People's Liberation Army 59th Hospital, China

## Abstract

Mutations in the gene* BEST1* usually cause bestrophinopathies, such as the rare progressive diseases Best vitelliform macular dystrophy (BVMD) and autosomal recessive bestrophinopathy (ARB). This study aimed to investigate the clinical characteristics of patients with BVMD or ARB carrying* BEST1* mutations. A total of 12 probands including 9 patients with a clinical diagnosis of BVMD and 3 patients with a clinical diagnosis of ARB were recruited for genetics analysis. All patients underwent detailed ophthalmic examination. All coding exons of the* BEST1* gene were screened by PCR-based DNA sequencing. Programs of PolyPhen-2, SIFT, and MutationTaster were used to analyze the potential pathogenicity of the mutations in* BEST1*. In the 9 unrelated patients with BVMD, one heterozygous* BEST1* mutation was revealed in 8 patients and two compound heterozygous mutations in 1 patient. In the 3 unrelated patients with ARB, two compound heterozygous mutations were revealed in 2 patients and three compound heterozygous mutations in 1 patient. Molecular analyses identified a total of 15 mutations, including 3 novel mutations (c.424A>G p.S142G, c.436G>A p.A146T, and c.155T>C p.L52P). Antivascular endothelial growth factor (VEGF) drugs were given to two affected eyes, especially those also exhibiting choroidal neovascularization (CNV), and no serious adverse events occurred. Our study indicates that there is wide genotypic and phenotypic variability in patients with BVMD or ARB in China. The screening of* BEST1 *gene is significant for the precise diagnosis of BVMD and ARB.

## 1. Introduction

Best vitelliform macular dystrophy (BVMD; OMIM153700) is a maculopathy characterized by the deposition of yellowish, lipofuscin-like or vitelliform lesions that often show considerable morphologic variability in different stages of the disease [1]. The lesion usually evolves through previtelliform, vitelliform, pseudohypopyon, vitelliruptive, and atrophic/cicatricial stages with time [2]. Electrooculography (EOG) with an Arden ratio of light-peak to dark-trough less than 1.5 is usually a specific clinical diagnostic indicator for BVMD [3]. BVMD is inherited in an autosomal dominant fashion but with variable expressivity [4]. The incidence was noted to be 1.5-20/100000 [5, 6]. The disease was first described by the physician Friedrich Best in 1905 [7].

As for autosomal recessive bestrophinopathy (ARB; OMIM611809), Schatz described a patient with yellowish vitelliform lesion identified with biallelic variants of the* BEST1 *gene in 2006 [8]. Burgess et al. first reported ARB as a distinct retinal disease associated with the* BEST1 *gene in 2008 [9]. As opposed to multifocal Best disease and classic Best disease which have autosomal dominant inheritance, the parents in many ARB cases do not have fundus findings and their EOG is normal [10]. OCT imaging highlights both the serous detachment and the hyperreflectivity of the vitelliform lesions. Some patients also present with a cystoid macular edema and, like the vitelliform lesions, the central scar is hyperreflective [1, 4]. It was reported that some cases were hyperopic or had shallow anterior chamber angles that predisposed them to angle-closure glaucoma [11]. EOG shows an absent or markedly reduced light-peak. Reduced amplitudes of electroretinograms (ERGs) are also associated with ARB [12]. However, ERG may remain relatively normal for a long time [11].

The* BEST1* gene (OMIM 607854, formerly named* VMD2*: OMIM 153700), which contains 11 exons and is located on chromosome 11q12-13, has been identified as the disease-causing gene for a variety of diseases called bestrophinopathies, such as BVMD, ARB, retinitis pigmentosa, and autosomal dominant vitreoretinochoroidopathy (ADVIRC) [13, 14]. It is expressed predominantly in retinal pigment epithelium (RPE), and the mRNA encodes the 585-amino acid protein bestrophin-1 [15]. Bestrophin-1 is presumed to function as a Cl- channel activated by Ca2+, an inhibitor of the intracellular voltage-dependent Ca2+ channel, and a channel that also transports HCO3- [16, 17]. More than 300 mutations have been identified to be associated with bestrophinopathies thus far.

Currently, there are no concrete treatments that halt the progressive maculopathy in bestrophinopathy [10]. Appropriate therapies like drug treatment and gene therapy to achieve a better prognosis for bestrophinopathy have been explored for decades [10]. In this study, we investigated the genotypes, clinical characteristics, and therapeutic options of patients with bestrophinopathy in our department to both better understand the disease and improve clinical management.

## 2. Methods

### 2.1. Patients and Clinical Data

The research received institutional approval by the Ethical Committee and Institutional Review Board of Peking University People's Hospital. The study conformed to the Declaration of Helsinki and was carried out in accordance with institutional guidelines. Informed consent was obtained from all participants enrolled in the study. In total, 12 unrelated Chinese patients were recruited in this study from 2012 to 2017. In this study, 4 sporadic patients and 8 patients with family members were analyzed. Detailed ophthalmic examination, including measurement of best corrected visual acuity (BCVA), slit-lamp biomicroscopy, dilated fundus examination, fundus photography, fundus autofluorescence (FAF) imaging, fluorescein angiography (FA), indocyanine green angiography (ICGA), and optical coherence tomography (OCT, Heidelberg Engineering, Heidelberg, Germany), was conducted. Ten patients underwent EOG examinations and 6 patients underwent full-field ERG. Clinical treatments were recorded during the follow-up visit.

The diagnoses of BVMD and ARB were based on a combination of genetic tests of* BEST1* and ophthalmic examination. BVMD was diagnosed as follows: juvenile-to-adult onset metamorphopsia or vision loss, macular lesion showing vitelliform, vitelliruptive, pseudohypopyon, atrophic or cicatricial changes, and an abnormal EOG Arden ratio below 1.5. ARB was diagnosed based on yellow deep retinal/retinal pigment epithelial deposits present and reduced light-rise on EOG, associated with reduced amplitudes of ERG and an autosomal recessive inheritance pattern.

### 2.2. Molecular Methods

Peripheral blood samples were collected from all probands and some of their family members for genetic analysis. Genomic DNA was extracted from the peripheral blood samples using an Agilent SureSelect Target Enrichment System Kit (Agilent, USA). Polymerase chain reaction (PCR) was performed using Goldstar Taq MasterMix (Cwbio, PRC) to amplify the exons of the* BEST1* gene (NM_004183.3). Samples were sequenced directly by loading the sequencing reaction product into NEXTSEQ500 (Illumina, USA).

The potential pathogenicity of novel missense mutations was investigated using the programs PolyPhen-2 (Polymorphism Phenotype; http://genetics.bwh.harvard.edu/pph/), SIFT (Sort Intolerant from Tolerant, http://sift.jcvi.org/), and MutationTaster (http://www.mutationtaster.org) as the standards and guidelines for the interpretation of sequence variants suggested by the American College of Medical Genetics and Genomics and the Association for Molecular Pathology in 2015. Finally, we verified the novel mutations using 100 heathy controls without any eye disease.

### 2.3. Statistical Methods

Results were expressed as frequencies and percentages for categorical variables and as mean ± SD for continuous variables.

## 3. Results

### 3.1. Mutation Analysis of the* BEST1* Gene

**Figure 1:**
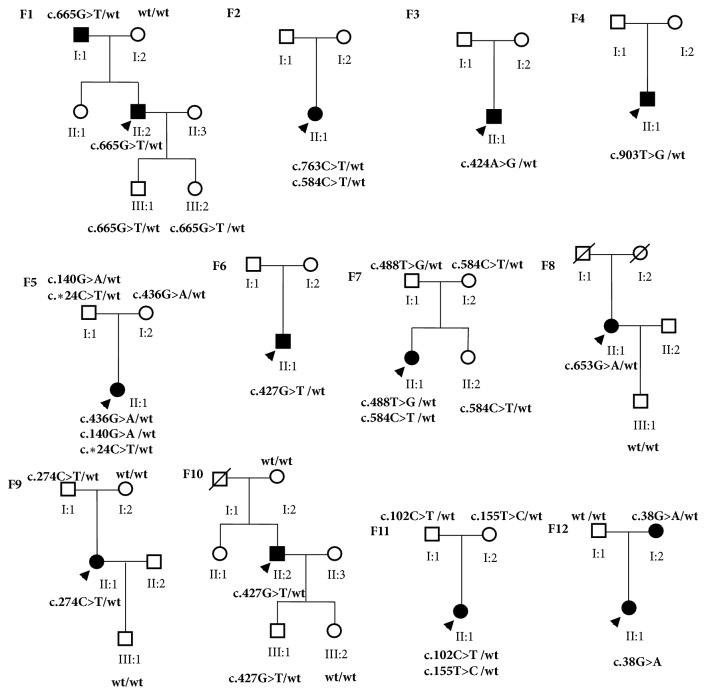
Pedigrees of families with* BEST1* mutations in this study. Blackened symbols: affected individuals; arrow below the symbol: the proband.

Direct sequencing analysis revealed a total of 15* BEST1* mutations, including 13 (86.7%) missense mutations, 1 (6.7%) splicing mutation c.*∗*24C>T, and 1 (6.7%) synonymous mutation c.102C>T/p.G34G (Table 1). Of these mutations, 8 different mutations were solely detected in patients with BVMD, 6 mutations were solely identified in patients with ARB, and one missense mutation (c.584 C>T p. A195V) was found in both patients with BVMD and those with ARB. In the 9 unrelated patients with BVMD, one heterozygous* BEST1* mutation was revealed in 8 patients and two compound heterozygous mutations in 1 patient. In the 3 unrelated patients with ARB, two compound heterozygous mutations were revealed in 2 patients and three compound heterozygous mutations in 1 patient. Pedigrees of families with* BEST1* mutations in this study were shown in Figure 1. Three missense mutations (c.424A>G p.S142G, c.436G>A p.A146T, and c.155T>C p.L52P) had not been previously reported in the literature or registered in the Ensembl database (http://www.ensembl.org/index.html) or the Human Gene Mutation Database (HGMD, http://www.hgmd.cf.ac.uk/ac/index.php). The missense mutation c.424A>G (p.S142G) was identified in BVMD patient without other mutation, and c.436G>A (p.A146T) and c.155T>C (p.L52P) were found in ARB patients combined with other* BEST1* mutations. None of the mutations were found in our 100 controls. The 3 novel mutations were predicted to be damaging by PolyPhen-2 (scores of 0.999, 1.000, and 0.794) and predicted to affect protein function by SIFT (scores of 0.00, 0.00, and 0.01). Program MutationTaster predicted that both the amino acid sequence and the splice site would be changed by the 3 novel mutations. And besides, protein features might be changed. Based on the ACMG standards and guidelines for the interpretation of sequence variants in 2015, the three novel missense mutations c.424A>G (p.S142G), c.436G>A (p.A146T), and c.155T>C (p.L52P) were all considered pathogenic.

### 3.2. Clinical Features of the Patients Carrying Different* BEST1* Mutations

**Figure 2:**
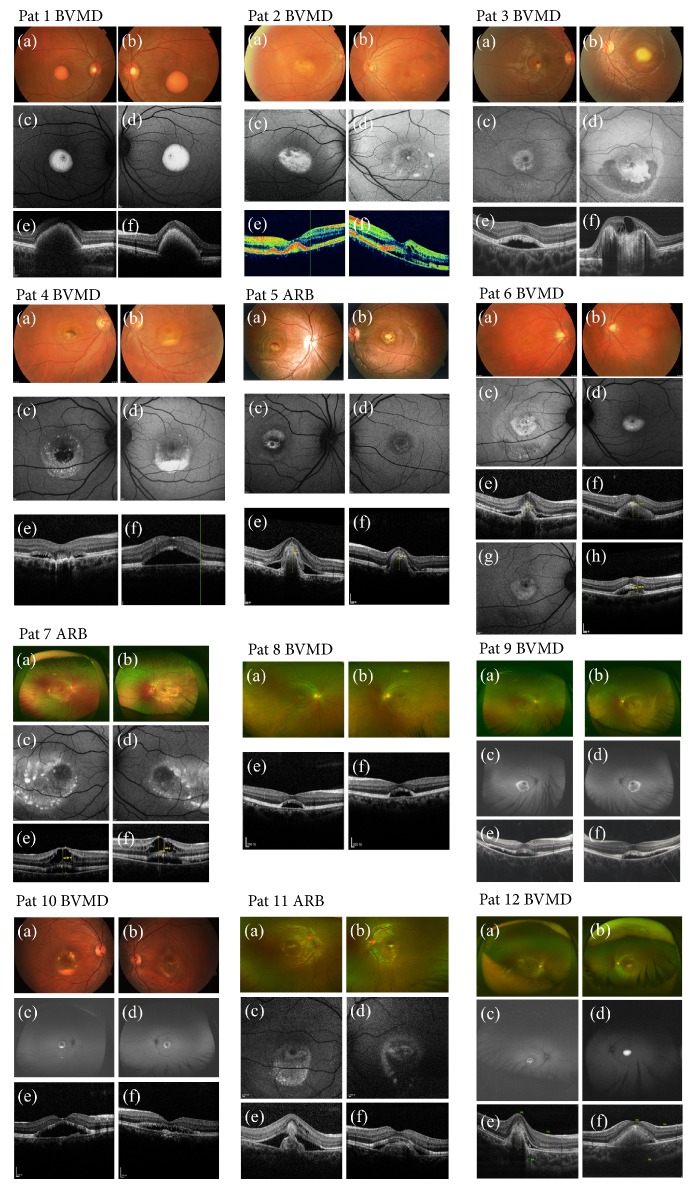
Clinical phenotypic images of the probands in this study. (a) OD fundus appearance. (b) OS fundus appearance. (c) OD FAF images. (d) OS FAF images. (e) OD macular OCT images. (f) OS macular OCT images. (g) OD FAF image of patient 6 after therapy of intravitreal injection of anti-VEGF. (h) OD OCT image of patient 6 after therapy of intravitreal injection of anti-VEGF.

The demographic and clinical information is listed in Tables 2 and 3. Data were collected from 2012 to 2017. Twelve probands from 12 unrelated Chinese families were recruited, and their clinical phenotypic images were shown in Figure 2. Nine patients, including 6 males and 3 females, were diagnosed with BVMD, and 3 female patients were diagnosed with ARB. The median age at onset of BVMD was 30 years (range, 4-49 years) and the median age at onset of ARB was 10 years (range, 5-11 years). The mean visual acuity (VA) was 0.48±0.35 in patients with BVMD (patients 2 and 4 were excluded, as their VA data were lost) and 0.35±0.21 in patients with ARB.

EOG was performed in 14 eyes of 7 patients with BVMD and 6 eyes of 3 patients with ARB (Table 4). The average EOG Arden ratio of light-peak to dark-trough was 1.130±0.247 (median 1.112, range 1.154-1.885) in BVMD eyes and 1.002±0.095 (median 0.981, range 0.911-1.112) in ARB eyes. The Arden radio was reduced (<1.5) in 13 BVMD eyes (13/14, 92.9%) and 6 ARB eyes (6/6, 100%). Six eyes of 3 BVMD patients (6/6, 100%) showed that absence of reduced amplitudes in ERG including the proband 1 identified the mutation c.665G>T (p.G222V) previously reported in leber congenital amaurosis. Three eyes of 3 ARB patients (3/6, 50%) showed reduced amplitudes of ERG.

Three eyes of 3 BVMD patients (3/18, 16.7%), whose ages were 8, 25, and 4, exhibited choroidal neovascularization (CNV) or fundus hemorrhage. Three eyes of 2 ARB patients (3/6, 50%) also exhibited CNV and 2 eyes of one patient (2/6, 33.3%) exhibited retinoschisis. Antivascular endothelial growth factor (VEGF) drugs, including bevacizumab, conbercept, and ranibizumab, were given to 3 eyes of 2 BVMD patients and 4 eyes of 2 ARB patients, especially those that also exhibited CNV or retinoschisis. Visual acuity was improved in 3 BVMD eyes (3/3) and 3 ARB eyes (3/4) after anti-VEGF therapy and no serious adverse events occurred. Two BVMD eyes accepted photodynamic therapy (PDT) with no adverse events, while the visual acuity declined from 0.5 to 0.4 after therapy.

### 3.3. Family Members

In total, 23 family members including 13 carriers underwent examinations, among which 2 family members with identified* BEST1* mutation were affected. For families 2, 3, 4, and 6, genetic data were available from one proband of each family. In family 1 (BVMD), a heterozygous c.665G>T (p.G222V) missense mutation was detected in 4 members. The proband's father (I-1) was found to have vitelliform changes at the macula, whereas his 4-year-old son (III-1) and 6-year-old daughter (III-2) had normal fundus appearances. In family 9 (BVMD), the fundus of the proband's father (I-1) with a heterozygous c.274C>T (p.R92C) mutation was found to be normal. In family 12 (BVMD), the proband's mother (I-2) with a heterozygous c.38G>A (p.R13H) mutation was found to have a reduced Arden ratio, while her fundus examination was normal. In family 10 (BVMD), the fundus of the proband's 8-year-son (III-1) with a heterozygous c.427G>T (p.V143F) mutation was found to be normal. The fundi of the other members without the mutation were all normal. In families 5, 7, and 11 (ARB), the parents with mutations were all found to have normal Arden ratio and normal fundus appearances. The latter three families showed recessive inheritance.

## 4. Discussion

The study confirms that* BEST1* gene mutation is the primary factor in the development of BVMD and ARB. In this study, we identified a novel missense mutation c.424A>G (p.S142G) in association with a BVMD proband and two novel missense mutations, c.436G>A (p.A146T) and c.155T>C (p.L52P), in association with 2 ARB probands. It is reported that more than 300 distinct* BEST1* mutations have been found in sporadic patients and families affected by various bestrophinopathies [18, 19]. In previous study in Chinese patients, many novel mutations have also been identified including c.763C>T (R255W) and c.488T>G (M163R) in this study [20, 21]. Here our findings expand the mutational spectrum of* BEST1 *and suggest that the mutations of* BEST1* in Chinese people may be different in comparison to other ethnic groups.

**Figure 3:**
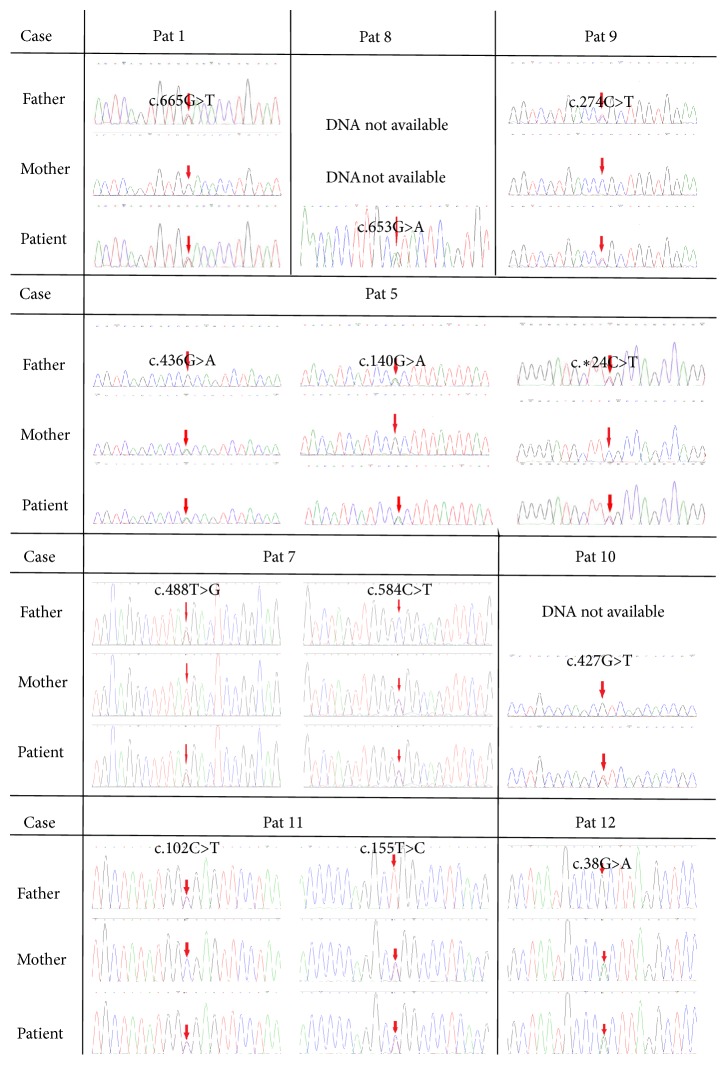
Sanger confirmation of the identified* BEST1* variants. Sequence chromatograms of patients and their parents (where available) are shown.

In total, 15 mutations in* BEST1* were found in this study. The known mutations reported were associated with leber congenital amaurosis (LCA), BVMD, adult vitelliform macular dystrophy (AVMD), or ARB [15, 22–27]. Sanger confirmation of the identified* BEST1* variants in probands with family members is shown in Figure 3. Of the 15 mutations, 8 were associated with BVMD, 6 were associated with ARB, and 1 mutation c.584C>T (p.A195R) was associated with BVMD and ARB. In our study, missense mutations were the leading cause of bestrophinopathy, accounting for 100% of all mutations identified in BVMD and 71.4% in ARB. It is noteworthy that the missense mutation c.424A>G (p.S142G) associated with a BVMD patient and the mutations c.436G>A (p.A146T) and c.155T>C (p.L52P) associated with 2 ARB patients were novel. None of the mutations were present in HGMD, the Ensembl database, and the genic testing of 100 healthy controls. The other mutations identified in the study have been previously reported in different countries such as America and China et al. [28, 29]. In previous reports, cases with compound heterozygous variants were reported, which was rare in BVMD [30, 31]. In our study, we found a patient with two compound heterozygous missense mutations and a typical phenotype for BVMD. The patient with identified BEST1 mutations c.763C>T (p.R255W) and c.584C>T (p.A195V) was a 34-year-old female. It was reported that c.763C>T (p.R255W) was identified in BVMD [20] and c.584C>T (p.A195V) was identified both in BVMD and ARB [23, 25]. There was absence of reduced amplitude of ERG in both eyes. The remaining 3 patients with compound* BEST1* mutations expressed the phenotype for ARB. In one of the patients three mutations were identified, among which the splicing mutation c.*∗*24C>T was considered to be benign. There is wide genetic and phenotypic variability in Chinese patients with BVMD and ARB.

Bestrophin-1 encoded by the* BEST1* gene is an integral membrane protein localized predominantly to the basolateral membrane of RPE [32]. Numerous missense mutations in genes encoding integral membrane proteins are related to defective cellular trafficking [33, 34]. A study investigated whether 13 missense mutations, located in four mutational hot-spots of bestrophin-1, affect plasma membrane targeting in polarized MDCK II cells [29]. All mutants mentioned in that study showed a significant reduction in anion conductance, indicating that disease-associated missense mutations in bestrophin-1 affect cellular trafficking and anion conductance and thus may be a common cause of bestrophinopathy. Similarly, it is reported that splicing mutations may interfere with exon splicing of mRNA, leading to an altered genic product [35].* BEST1* knock-in mice model has been produced and exhibited a phenotype similar to BVMD [36]. Compared to BVMD, ARB has been theorized to be associated with a null phenotype for* BEST1*. However, the* BEST1* knock-out mice model did not exhibit bestrophinopathy [37]. Thus whether ARB is a true “null” phenotype needs to be explored. Anyhow, further studies on the effects of the mutation on cellular function are necessary.

Our study included 9 probands from 9 families diagnosed with BVMD and 3 probands from 9 families diagnosed with ARB. The median age at onset was 30 years (range 4-49) in BVMD and 10 years (5-11) in ARB. The age of BVMD at onset varied widely which was similar to previous report, while ages at onset of our ARB probands were relatively young [11]. The mean VA was 0.48±0.35 in patients with BVMD and 0.35±0.21 in patients with ARB. The average EOG Arden ratio was 1.130±0.247 in BVMD and 1.002±0.095 in ARB, which were obviously reduced compared to normal values. Eyes with reduced Arden radio (<1.5) composed large proportions of 92.9% (13/14) in the BVMD sample and 100% (6/6) in the ARB sample. EOG of the right eye in a BVMD patient was normal, and the patient was an 8-year-old male with an identification of* BEST1* mutation c.424A>G (p.S142G). The reason why this mutation did not cause reduced Arden ratio of EOG in one eye needs to be explored. EOG evaluates the function of the outer retina and RPE using the RPE's response to changing illumination [38]. The result infers that the function of RPE cells was impaired in the development of most BVMD and ARB.

There is no concrete treatment for a clinical patient suffering from bestrophinopathy at the moment. Studies in the laboratory suggested that treatment with valproic acid was able to increase the rate of photoreceptor outer segment degradation in an iPSC-RPE model through changing the level of exosome secretion, protein oxidation, and free-ubiquitin [39]. Our goal of therapy was controlling the CNV or retinal hemorrhage secondary to BVMD or ARB. In our study, a patient (patient 6) had CNV secondary to BVMD in the right eye. The VA was 0.25 on initial presentation. After acceptance of intravitreal injection of bevacizumab (IVB) 3 times and conbercept (IVC) and ranibizumab (IVR) once, his BCVA improved to 1.0 while CNV was reduced according to FAF and OCT examinations (Figure 2  (g), (h)). There were no complications after the therapy during the two-year follow-up. In total, anti-VEGF drugs, including bevacizumab, conbercept, and ranibizumab, were given to 3 eyes of 2 BVMD patients and 4 eyes of 2 ARB patients, especially those that also exhibited CNV or retinoschisis. Visual acuity was improved in 3 BVMD eyes (3/3) and 3 ARB eyes (3/4) after anti-VEGF therapy and no serious adverse events occurred. Similarly, previous study also described patient with CNV caused by BVMD who obtained good visual acuity after anti-VEGF treatment [40]. The outcome suggests that anti-VEGF drugs may be an appropriate way to control CNV secondary to bestrophinopathy.

Several gene therapy trials in the retina are currently underway and gene therapy has been shown to be effective for some diseases such as RPE65-Leber congenital amaurosis [41]. Gene therapy in animal models will provide a basis for gene-directed therapy. Our study provided data on genetic mutations and clinical features to assist the exploration of gene therapy in patients with bestrophinopathy. However, there are still many challenges in gene therapy, including an appropriate time of gene intervention and the management of complications and future adverse events during the course of treatment.

In conclusion, we found a novel causative missense mutation, c.424A>G (p.S142G), associated with BVMD and two novel causative missense mutations, c.436G>A (p.A146T) and c.155T>C (p.L52P), associated with ARB. Management of CNV secondary to BVMD or ARB should be taken into consideration to prevent progression and improve visual acuity. Our results expand the data on the mutational findings and clinical characteristics of the disease. We believe that our work will facilitate the diagnosis, clinical therapy, and genetic counseling of BVMD and ARB. Furthermore, we will explore the concrete pathogenesis of the mutations in this investigation that caused BVMD and ARB in cell and animal model in a future study.

## Figures and Tables

**Table 1:** Mutations of *BEST1* genetic testing results^1^.

**Patient Number**	**Position**	**Nucleotide Change**	**Amino Acid Change**	**Mutation Type**	**Novel**	**Phenotype**	**Disease Reported**	**Population Frequency**
1	Exon 6	c.665G>T	G222V	Missense	No	BVMD	LCA	absent

2	Exon 7	c.763C>T	R255W	Missense	No	BVMD	BVMD	0.0000505
	Exon 5	c.584C>T	A195V	Missense	No		BVMD ARB	0.0002381

3	Exon 4	c.424A>G	S142G	Missense	Yes	BVMD	-	absent

4	Exon 8	c.903T>G	D301E	Missense	No	BVMD	BVMD	absent

5	Exon 4	c.436G>A	A146T	Missense	Yes	ARB	-	absent
	Exon 2	c.140G>A	R47H	Missense	No		AVMD	0.00001626
	3 prime UTR	c.*∗*24C>T	-	Splicing	No		Not provided	0.0002208

6	Exon 4	c.427G>T	V143F	Missense	No	BVMD	AVMD	absent

7	Exon 5	c.488T>G	M163R	Missense	No	ARB	ARB	absent
	Exon 5	c.584C>T	A195V	Missense	No		BVMD	0.0002381

8	Exon 6	c.653G>A	R218H	Missense	No	BVMD	BVMD	0.00001218

9	Exon 4	c.274C>T	R92C	Missense	No	BVMD	BVMD	absent

10	Exon 4	c.427G>T	V143F	Missense	No	BVMD	AVMD	absent

11	Exon 2	c.102C>T	G34G	Synonymous	No	ARB	ARB	0.00001627
	Exon 3	c.155T>C	L52P	Missense	Yes		-	absent

12	Exon 2	c.38G>A	R13H	Missense	No	BVMD	BVMD	0.00001445

^1^NM_004183.3.UTR: untranslated region. Blank section means no data available. LCA: leber congenital amaurosis. BVMD: best vitelliform macular dystrophy. AVMD: adult vitelliform macular dystrophy. ARB: autosomal recessive bestrophinopathy.Population frequency was acquired in gnomAD database.

**Table 2:** Clinical data of patients with BVMD.

**Patient ** **Number**	**Gender**	**Age ** **(years)**	**Phenotype**	**Stages**		**Therapies**	**VA (first visit)**		**VA (after therapies)**	
				**OD**	**OS**		**OD**	**OS**	**OD**	**OS**
1	Male	30	BVMD	vitelliform	vitelliform	-	0.8	0.12	-	-

2	Female	34	BVMD	vitelliform	vitelliruptive	-	defect	defect	-	-

3	Male	8	BVMD with OS CNV	vitelliruptive	atrophic/cicatricial	OS IVB*∗*1	0.6	0.07	0.6	0.1

4	Male	20	BVMD	vitelliruptive	pseudohypopyon	-	defect	defect	-	-

6	Male	25	BVMD with OD CNV	atrophic/cicatricial	vitelliruptive	OD IVB*∗*3/IVC *∗*1/IVR*∗*1 OS IVC*∗*1	0.25	0.8	1.0	1.0

8	Female	49	BVMD	vitelliruptive	vitelliruptive	OU PDT*∗*1	0.5	0.5	0.4	0.4

9	Female	30	BVMD	vitelliruptive	vitelliruptive	-	1.0	1.2	-	-

10	Male	38	BVMD	pseudohypopyon	vitelliruptive	-	0.25	0.25	-	-

12	Male	4	BVMD with OD fundus hemorrhage	atrophic/ cicatricial	vitelliform	-	0.075	0.4	-	-

BVMD: best vitelliform macular dystrophy. VA: visual acuity. OD: oculus dexter, right eye. OS: oculus sinister, left eye. OU: oculus uterque, binoculus. CNV: choroidal neovascularization. IVB: intravitreal injection of bevacizumab. IVC: intravitreal injection of conbercept. IVR: intravitreal injection of ranibizumab. PDT: photodynamic therapy. Asterisks mean times of therapy. Blank sections mean no data available.

**Table 3:** Clinical data of patients with ARB.

**Patient Number**	**Gender**	**Age (years)**	**Phenotype**	**Therapies**	**VA (first visit)**		**VA (after therapies)**	
					**OD**	**OS**	**OD**	**OS**
5	Female	10	ARB with OU CNV	OD IVB*∗*2 OS IVB*∗*1	0.05	0.6	0.2	0.63
7	Female	11	ARB with OU retinoschisis	OU IVR*∗*1	0.5	0.5	0.5	0.63
11	Female	5	ARB with OD CNV	-	0.2	0.25	-	-

ARB: autosomal recessive bestrophinopathy. VA: visual acuity. OD: oculus dexter, right eye. OS: oculus sinister, left eye. OU: oculus uterque, binoculus. CNV: choroidal neovascularization. IVB: intravitreal injection of bevacizumab. IVR: intravitreal injection of ranibizumab. Asterisks mean times of therapy. Blank sections mean no data available.

**Table 4:** The data of the EOG Arden ratio of BVMD or ARB patients.

Patient Number	Phenotype	EOG (Arden ratio)	
		OD	OS
1	BVMD	1.154	1.204

2	BVMD	1.100	1.037

3	BVMD	1.885	0.892

5	ARB	0.922	1.038

6	BVMD	1.127	1.153

7	ARB	0.924	0.911

8	BVMD	1.105	1.119

9	BVMD	0.961	0.902

10	BVMD	0.910	1.284

11	ARB	1.107	1.112

EOG: electrooculogram.

## Data Availability

The data used to support the findings of this study are available from the corresponding author upon request.
